# Lipopolysaccharide inhibits myogenic differentiation of C2C12 myoblasts through the Toll-like receptor 4-nuclear factor-κB signaling pathway and myoblast-derived tumor necrosis factor-α

**DOI:** 10.1371/journal.pone.0182040

**Published:** 2017-07-24

**Authors:** Yuko Ono, Kazuho Sakamoto

**Affiliations:** 1 Department of Pharmacology, School of Medicine, Fukushima Medical University, Fukushima, Japan; 2 Emergency and Critical Care Medical Center, Fukushima Medical University Hospital, Fukushima, Japan; University of Minnesota Medical Center, UNITED STATES

## Abstract

**Background:**

Circulating lipopolysaccharide (LPS) concentrations are often elevated in patients with sepsis or with various endogenous diseases that are associated with metabolic endotoxemia. Involuntary loss of skeletal muscle, termed muscle wasting, is commonly observed in these conditions, suggesting that circulating LPS might play an essential role in its development. Although impairment of muscle regeneration is an important determinant of skeletal muscle wasting, it is unclear whether LPS affects this process and, if so, by what mechanism. Here, we used the C2C12 myoblast cell line to investigate the effects of LPS on myogenesis.

**Methods:**

C2C12 myoblasts were grown to 80% confluence and induced to differentiate in the absence or presence of LPS (0.1 or 1 μg/mL); TAK-242 (1 μM), a specific inhibitor of Toll-like receptor 4 (TLR4) signaling; and a tumor necrosis factor (TNF)-α neutralizing antibody (5 μg/mL). Expression of a skeletal muscle differentiation marker (myosin heavy chain II), two essential myogenic regulatory factors (myogenin and MyoD), and a muscle negative regulatory factor (myostatin) was analyzed by western blotting. Nuclear factor-κB (NF-κB) DNA-binding activity was measured using an enzyme-linked immunosorbent assay.

**Results:**

LPS dose-dependently and significantly decreased the formation of multinucleated myotubes and the expression of myosin heavy chain II, myogenin, and MyoD, and increased NF-κB DNA-binding activity and myostatin expression. The inhibitory effect of LPS on myogenic differentiation was reversible, suggesting that it was not caused by nonspecific toxicity. Both TAK-242 and anti-TNF-α reduced the LPS-induced increase in NF-κB DNA-binding activity, downregulation of myogenic regulatory factors, and upregulation of myostatin, thereby partially rescuing the impairment of myogenesis.

**Conclusions:**

Our data suggest that LPS inhibits myogenic differentiation via a TLR4–NF-κB-dependent pathway and an autocrine/paracrine TNF-α-induced pathway. These pathways may be involved in the development of muscle wasting caused by sepsis or metabolic endotoxemia.

## Introduction

Lipopolysaccharide (LPS), the major molecular component of the outer membrane of gram-negative bacteria, binds to Toll-like receptor 4 (TLR4) and induces formation of a TLR4–CD14 complex that increases nuclear factor-κB (NF-κB) activity [1,2]. LPS can cause a dysregulated inflammatory response leading to life-threatening organ dysfunction; a syndrome termed sepsis [3]. Increased levels of circulatory LPS are observed in patients with sepsis [4], elderly subjects [5,6] and individuals with diabetes mellitus [7], obesity [7], human immunodeficiency virus infection [8,9], cancer [10,11], liver cirrhosis [12], and end-stage kidney disease [13,14]. In the latter cases, increased LPS levels are caused by bacterial translocation from the intestinal tract to the circulation [15], a phenomenon known as metabolic endotoxemia [15]. Severe involuntary loss of skeletal muscle, termed muscle wasting, can be observed in all of these conditions [16], suggesting a potential role for circulating LPS in its development. Muscle wasting contributes to generalized weakness and debilitation, worsens quality of life, and increases mortality and economic burden [17]. Thus, there is an urgent need to advance our knowledge of its molecular pathogenesis.

One important cause of muscle wasting is breakdown of muscle protein through the ubiquitin–proteasome-dependent pathway [18]. Previous studies have shown that LPS activates the ubiquitin–proteasome pathway through TLR4 and induces catabolism both in cultured C2C12 muscle cells [19] and in rat muscle in vivo [20]. In agreement with these findings, increased ubiquitin–proteasome activity has been reported in elderly subjects [21] and in patients with metabolic endotoxemia due to diabetes mellitus [22], obesity [23], liver cirrhosis [24], and chronic kidney disease [25,26]. Damaged or degenerated myofibers are repaired or replaced through myogenesis, the process by which myoblasts fuse to form multinucleated myotubes. Although reduced myogenic capacity is another important determinant of skeletal muscle wasting [27–31], it is not known whether LPS affects this process.

Vertebrate skeletal muscle myogenesis is under the strict control of muscle-specific transcription factors such as MyoD and myogenin [32,33] and negative regulatory factors such as myostatin [34–36]. Previous work with cultured C2C12 myoblasts suggests that exogenous tumor necrosis factor α (TNF-α) inhibits myoblast differentiation by downregulating myogenin and MyoD via NF-κB activation [27–31]. Hyperammonemia [37] and reactive oxygen species [38] also act through NF-κB to induce myostatin expression in mouse myoblasts. Whether and how LPS affects myogenesis regulatory factors is unknown. Since TLR4 is expressed in skeletal muscle [39–41] and circulating LPS can reach peripheral tissues [42], we hypothesized that LPS might perturb both positive and negative regulatory factors via TLR4–NF-κB signaling in differentiating myoblasts, thereby suppressing muscle regeneration.

LPS stimulates expression of proinflammatory cytokines, including TNF-α, not only in classical immune tissues but also in skeletal muscle [40,41,43]. Since TNF-α contributes to many pathogenic processes, including insulin resistance [44,45] and carcinogenesis [46], through both autocrine and paracrine mechanisms, it is possible that LPS-induced TNF-α secretion by myoblasts might also play a role in muscle wasting.

Here, we aimed to evaluate the effect of LPS on myogenesis, including the possible roles of TLR4–NF-κB signaling and autocrine/paracrine TNF-α on both positive and negative muscle regulatory factors. We found that selective inhibition of TLR4 signaling or neutralization of TNF-α activity had a beneficial effect on LPS-treated C2C12 myoblasts. Thus, TLR4–NF-κB signaling and myoblast-derived TNF-α play key roles in the impairment of muscle regeneration.

## Materials and methods

### Myogenic cell culture

The murine C2C12 myoblast cell line was obtained from the RIKEN Cell Bank (Cell No. RCB0987, Tsukuba, Japan). Myoblasts were cultured in growth medium consisting of high-glucose Dulbecco’s modified Eagle’s medium (DMEM; Wako, Osaka, Japan), 10% (vol/vol) fetal bovine serum (Equitech Bio, Kerrville, TX), 100 U/mL penicillin, and 100 μg/mL streptomycin (Wako) at 37°C in a humidified atmosphere of 5% CO_2_. When the cells reached 80% confluence, the culture medium was changed to differentiation medium (DM), consisting of high-glucose DMEM, 2% heat-inactivated horse serum (Thermo Fisher Scientific, Waltham, MA), 100 U/mL penicillin, and 100 μg/mL streptomycin, to induce myogenesis.

C2C12 myoblasts were treated with LPS from *Escherichia coli* 026:B6 (Sigma Aldrich, St. Louis, MO) dissolved in phosphate-buffered saline (PBS) at a concentration of 0.1 or 1 μg/mL for various times between 2 h and 144 h, as indicated. MyoD and myogenin expression and NF-κB (p65) DNA-binding activity were analyzed at 48 h, while expression of myosin heavy chain (MyHC) II, a myogenic marker, and myostatin was measured at 144 h. The TLR4 signaling inhibitor TAK-242 (Merck Millipore, Darmstadt, Germany) was added to cells at a final concentration of 1 μg/mL in dimethyl sulfoxide (DMSO, 0.1% vol/vol) immediately after the addition of LPS. The anti-TNF-α-neutralizing antibody (goat polyclonal, Cat. No. AB-410-NA; R&D Systems, Minneapolis, MN) was added at 5 μg/mL in PBS at 1 h prior to the addition of LPS. The anti-TLR2 neutralizing antibody T2.5 (mouse monoclonal, Cat. No. mab-mtlr2; InvivoGen, San Diego, CA) was added at 10 μg/mL in PBS at 1 h prior to the addition of LPS. The cell stimulators and inhibitors were present throughout the incubation. DM containing the appropriate concentrations of vehicle, LPS, TAK-242, and antibodies was exchanged every other day.

### Total RNA isolation and reverse-transcription polymerase chain reaction (RT-PCR)

Total RNA was extracted from C2C12 cells using ISOGEN (Wako) according to the manufacturer’s protocol. Precipitated total RNA was dissolved in diethylpyrocarbonate-treated water. To remove contaminating genomic DNA, the samples were treated with recombinant DNase I (Takara Bio, Kusatsu, Japan) for 15 min at 37°C and re-precipitated. First-strand cDNA was prepared from total RNA (1 μg) using random hexamer priming and Moloney murine leukemia virus reverse transcriptase (Thermo Fisher Scientific) in a final reaction volume of 20 μL. The cDNA was diluted 5-fold with water and used as a template for PCR analysis. Primer sequences for murine TLR4, glyceraldehyde 3-phosphate dehydrogenase (GAPDH), and casein kinase 2a2 (CK2) were: TLR4 Fw: 5′-CAAGAACATAGATCTGAGCTTCAACCC-3′ and Rv: 5′-GCTGTCCAATAGGGAAGCTTTCTAGAG-3′; GAPDH Fw: 5′-ACCACAGTCCATGCCATCAC-3′ and Rv: 5′-CACCACCCTGTTGCTGTAGCC-3′; and CK2 Fw: 5′-GGAGGCCCTAGATCTTCTTG-3′ and Rv: 5′-CGCGTTAAGACGTTTTGATT-3′. PCR was carried out with 35 amplification cycles and an annealing temperature of 58°C using a MyCycler Thermal Cycler (Bio-Rad Laboratories, Hercules, CA) and Taq DNA polymerase (Takara Bio). The PCR products were separated by 1.5% agarose gel electrophoresis and visualized by ethidium bromide staining.

Quantitative analysis of TLR4 transcripts was performed in duplicate by real-time RT-PCR using SYBR Premix Ex Taq (Takara Bio), reaction capillaries (Roche Diagnostics, Mannheim, Germany), and a Light Cycler 1.5 (Roche Diagnostics). CK2 transcripts were quantified as an internal control. Real-time RT-PCR was performed as described previously [47,48], and included an initial denaturing step at 95°C for 30 s; 40 cycles of denaturing at 95°C for 5 s, annealing at 58°C for 10 s, and extension at 72°C for 15 s; and a final heating step for dissociation analysis. Crossing points were determined by the second derivative maximum method [49], and expression levels were calculated by a modified version of the standard curve method [50] using Light Cycler software version 3.5 (Roche Diagnostics).

### Histological assessment

Cells were subjected to May–Grünwald and Giemsa staining to allow clear visualization of nuclei and myotube structures for quantitative measurements. After incubation in DM for 144 h, C2C12 cells were washed in cold PBS, fixed in 100% methanol, and stained as previously described [51] with minor modifications. Briefly, May–Grünwald staining solution (Wako) was diluted 1:3 in sodium phosphate buffer (1 mM NaH_2_PO_4_•H_2_O and 1 mM Na_2_HPO_4_, pH 6.0) and added to the cells for 5 min. Cells were then washed in distilled water and incubated in Giemsa staining solution (Wako) diluted 1:10 in distilled water for 10 min. Finally, cells were washed twice with distilled water and visualized with an inverted microscope (Olympus CK40, Tokyo, Japan) equipped with a camera (Olympus DP21). The myogenic index [52,53] was used as a morphological parameter of muscle differentiation. The number of nuclei in each myotube containing ≥3 nuclei and the total number of nuclei were counted in 5 randomly selected fields per well. The myogenic index (in %) was then calculated as: ([number of nuclei in myotubes in 5 fields] / [total number of nuclei in 5 fields] × 100) using ImageJ software version 1.39 (National Institutes of Health, Bethesda, MD). A total of 50–60 fields from 10–12 independent experiments was evaluated for each treatment group. Myotube widths were measured with ImageJ software using a modification of a published method [54–56]. In brief, cells were evaluated in 5 randomly selected fields per well. The width of each myotube containing ≥3 nuclei was measured at 3 different points on the cell and the average width per myotube was calculated. A total of 175–296 myotubes (50–60 fields, 10–12 independent experiments) was evaluated for each treatment group.

### Western blot analysis

Cells were washed twice in cold PBS and lysed in ice-cold radioimmunoprecipitation assay buffer consisting of 50 mM Tris-HCl (pH 8.0), 150 mM NaCl, 1% Nonidet P-40, 0.1% sodium dodecyl sulfate, and 0.5% sodium deoxycholate supplemented with 1% protease inhibitor cocktail (Thermo Fisher Scientific). Cell lysates were incubated on ice for 10 min, sonicated twice for 5 s each, and centrifuged at 4°C for 10 min at 15,000 *g*. The supernatants were collected and protein concentrations were determined using a protein assay kit (Bio-Rad Laboratories) with bovine serum albumin (Wako) as a standard. Equal amounts of protein (10 μg) per lane were resolved by 10% polyacrylamide gel electrophoresis, and proteins were transferred to a polyvinylidene difluoride membrane (Merck Millipore) using a wet transfer method and an XCell SureLock System (Thermo Fisher Scientific). The membrane was blocked in 5% (wt/vol) non-fat dried milk for 1 h at room temperature and washed in PBS-Tween 20 (0.1% vol/vol). Membranes were then incubated for 1 h at room temperature with antibodies specific for myosin heavy chain (MyHC) II (mouse monoclonal, Cat. No. 14–6503; Affymetrix, San Diego, CA. 1:200 dilution), MyoD (mouse monoclonal, sc-32758; Santa Cruz Biotechnology, Santa Cruz, CA. 1:100 dilution), myogenin (mouse monoclonal, sc-12732; Santa Cruz Biotechnology. 1:100 dilution), myostatin (rabbit polyclonal, ab71808; Abcam, Cambridge, UK. 1:500 dilution), or β-tubulin (rabbit polyclonal, ab6046; Abcam. 1:1000 dilution). After 3 washes of 10 min each, the blots were probed with a horseradish peroxidase-conjugated secondary antibody (goat anti-rabbit IgG, sc-2004 [Santa Cruz Biotechnology], 1:5000 dilution; or goat anti-mouse IgG, 62–6520 [Thermo Fisher Scientific], 1:3000 dilution) as appropriate. Densitometric analysis of protein bands was performed using ChemiDoc XRS Plus image analysis software (Bio-Rad Laboratories).

### NF-κB assay

NF-κB (p65) DNA-binding activity was determined using a TransAM enzyme-linked immunosorbent assay (ELISA) kit (Active Motif, Carlsbad, CA) according to the manufacturer’s protocol. In brief, nuclear extracts of C2C12 cells were prepared using the Nuclear Extract Kit (Active Motif), added to the oligonucleotide-coated plate, and incubated with the NF-κB p65-specific primary antibody (1:1000 dilution) and horseradish peroxidase-conjugated secondary antibody (1:1000 dilution) contained in the ELISA kit. After washing, the colorimetric reaction reagents were added, and sample absorbance at 450 nm was measured in a spectrophotometer (Multiskan GO, Thermo Fisher Scientific). The assay was performed in duplicate.

### Statistical analysis

The data are presented as the means ± standard error of the mean (SEM) unless otherwise indicated. Data were analyzed by one-way analysis of variance (ANOVA), and the treatment groups were compared with Tukey’s post hoc test for honest significant difference. All statistical analyses were performed using IBM SPSS Statistics for Windows, version 21.0 (IBM Corp., Armonk, NY). A *p* value <0.05 was considered statistically significant.

## Results

### TLR4 mRNA is expressed constitutively in differentiating myoblasts

**Fig 1:**
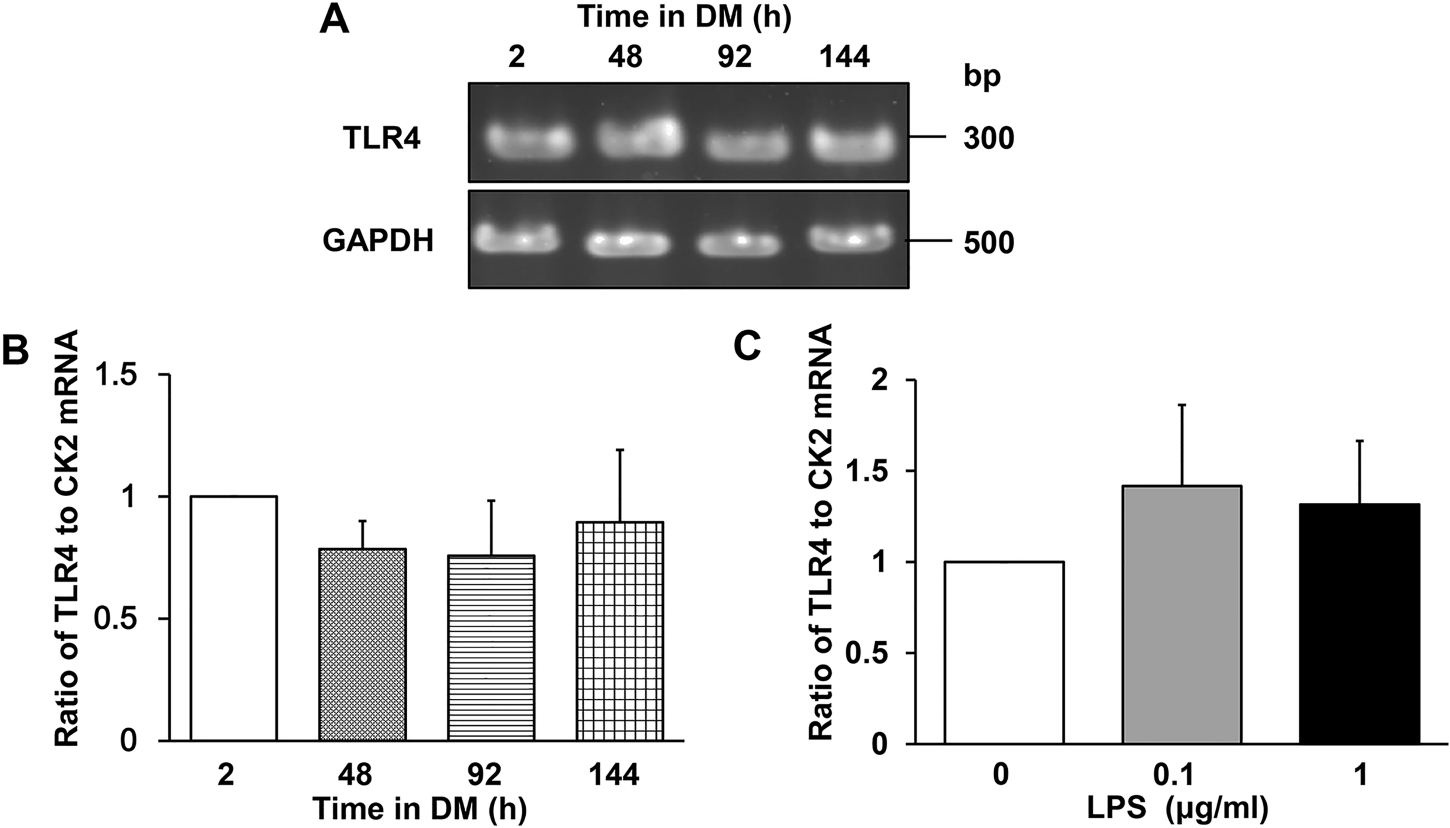
TLR4 mRNA expression in differentiating C2C12 myoblasts. (A–C) TLR4 mRNA levels in C2C12 myoblasts cultured in DM for 2, 48, 96, or 144 h with or without LPS. (A) Agarose gel of TLR4 and GAPDH (internal standard) mRNA levels during differentiation. (B) Real-time RT-PCR quantification of TLR4 mRNA levels. Data were normalized to CK2 mRNA levels and the ratio in cells cultured in DM for 2 h was set at 1.0. Data are the mean ± SEM of 3–4 independent experiments, each performed in duplicate. (C) Quantification of TLR4 mRNA by real-time RT-PCR. C2C12 cells were cultured with the indicated concentrations of LPS for 144 h. Data were normalized to CK2 mRNA levels and the ratio in untreated cells was set at 1.0. Data are the mean ± SEM of 3 independent experiments performed in duplicate. In (B) and (C), P > 0.05 for all comparisons.

Although TLR4 is abundantly expressed in skeletal muscle of various vertebrates, including human and mouse [39–41], its expression pattern in differentiating myoblasts is unclear. We examined TLR4 mRNA expression at 2, 48, 92, and 144 h after induction of C2C12 differentiation and found that it is expressed constitutively (Fig 1A). Quantitation of TLR4 mRNA by real-time RT-PCR confirmed that similar levels were expressed throughout the differentiation period (Fig 1B). Similarly, TLR4 mRNA levels did not change after culture of C2C12 cells in DM with LPS (0.1 or 1 μg/mL) for 144 h (Fig 1C), in agreement with previous observations by Frost et al. [43,57] and Lang et al. [41]. These results indicate that TLR4 would be expected to be available for LPS binding throughout C2C12 myogenesis and was unaffected by the presence of LPS.

### LPS inhibits myogenesis in a dose-dependent manner

**Fig 2:**
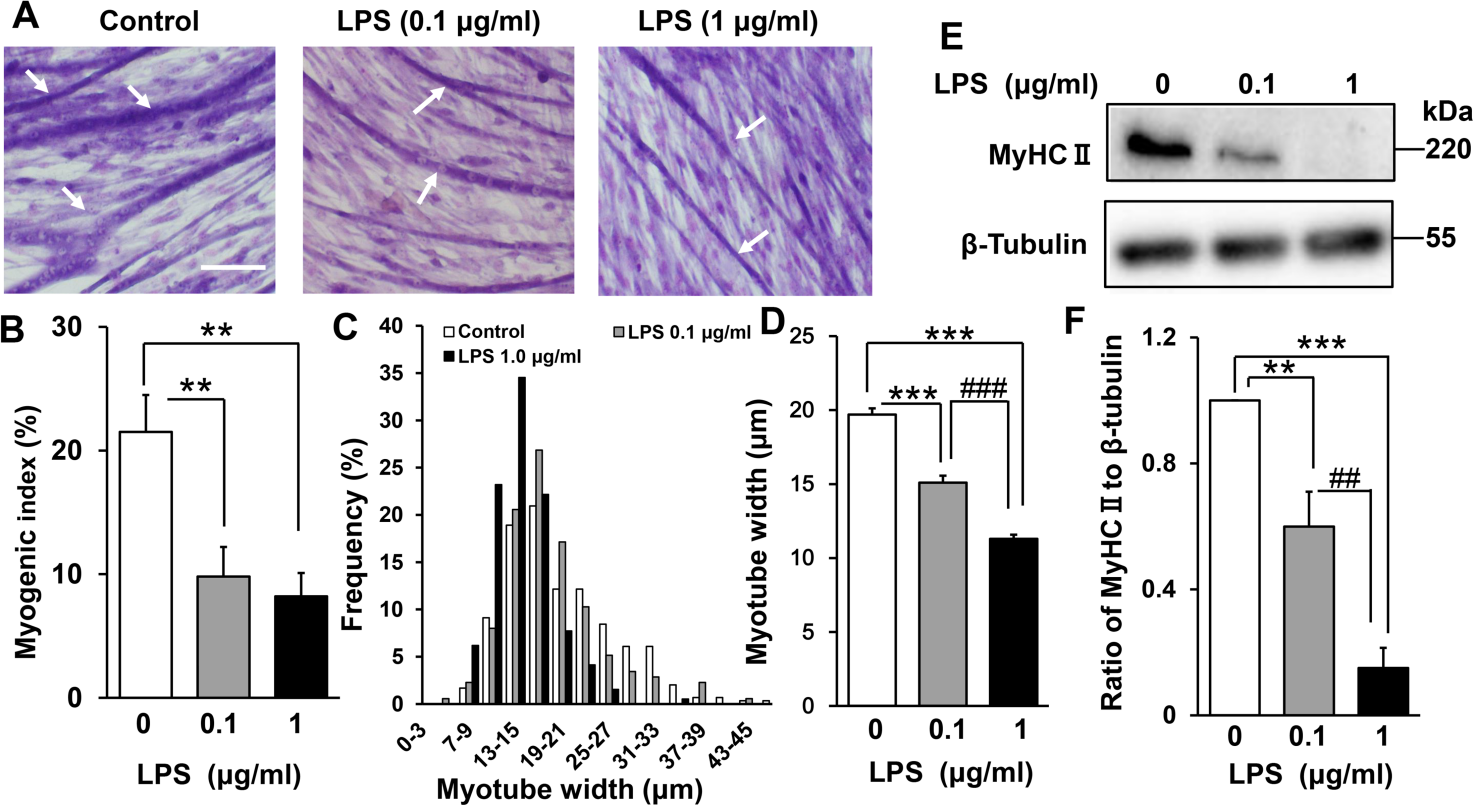
Effect of LPS on C2C12 myogenesis. (A) LPS inhibits the formation of myotubes. Myoblasts were cultured for 144 h in DM alone (control), DM plus LPS 0.1 μg/mL, or DM plus LPS 1 μg/mL, then fixed and subjected to May–Grünwald–Giemsa staining. Representative images are shown. Scale bar = 100 μm. Arrows indicate differentiated myotubes. (B) LPS decreases the myogenic index. Cells were treated as described in (A). Data are the mean ± SEM of 10–12 independent experiments, each examining 5 randomly selected fields (total 50–60 fields per treatment group). (C and D) LPS induces myotube atrophy. Cells were treated as described in (A), and the distribution of myotube widths (C) and mean myotube widths (D) were calculated. Data are the mean ± SEM of 10–12 independent experiments, each examining 5 randomly selected fields (total 175–296 myotubes from 50–60 fields per treatment group). (E) Representative western blot probed with antibodies to MyHC II or β-tubulin (internal standard). Cells were treated as described in (A). (F) Quantification of the data presented in (E). Data are the mean ± SEM of 13–14 independent experiments. ***p < 0.001, **p < 0.01, ###p < 0.001, ##p < 0.01 by one-way ANOVA followed by Tukey’s honest significant difference test.

To examine the effect of LPS on myogenesis, C2C12 cells were cultured in DM for 144 h in the presence or absence of 0.1 or 1 μg/mL LPS. These concentrations were employed for consistency with a previous study [19] that showed that LPS induces protein breakdown via the ubiquitin–proteasome pathway in differentiated C2C12 cells. LPS was found to inhibit the formation of multinucleated myotubes (Fig 2A). To quantify the morphological changes, the proportion of total nuclei that were present in myotubes was calculated (the myogenic index; see Materials and Methods). As shown in Fig 2B, the myogenic index was significantly lower in LPS-treated than untreated myoblasts (control, 21 ± 3%; LPS 0.1 μg/mL, 10 ± 2%; LPS 1 μg/mL, 8 ± 2%). LPS also dose-dependently decreased the average myotube width (control, 20 ± 0.4 μm; LPS 0.1 μg/mL, 15 ± 0.5 μm; LPS 1 μg/mL, 11 ± 0.3 μm; Fig 2C and 2D), in agreement with earlier observations that LPS induces muscle proteolysis [19]. We also confirmed by western blotting that LPS dose-dependently suppressed expression of the differentiation marker MyHC II (LPS 0.1 μg/mL vs control, 44% decrease [p < 0.01]; LPS 1 μg/mL vs control, 85% decrease [p < 0.001]; Fig 2E and 2F). Thus, LPS directly inhibits C2C12 myogenesis in a dose-dependent manner in the absence of immune cells, which are the major sources of inflammatory cytokines.

### Effect of LPS on muscle regulatory factors and NF-κB DNA-binding activity

**Fig 3:**
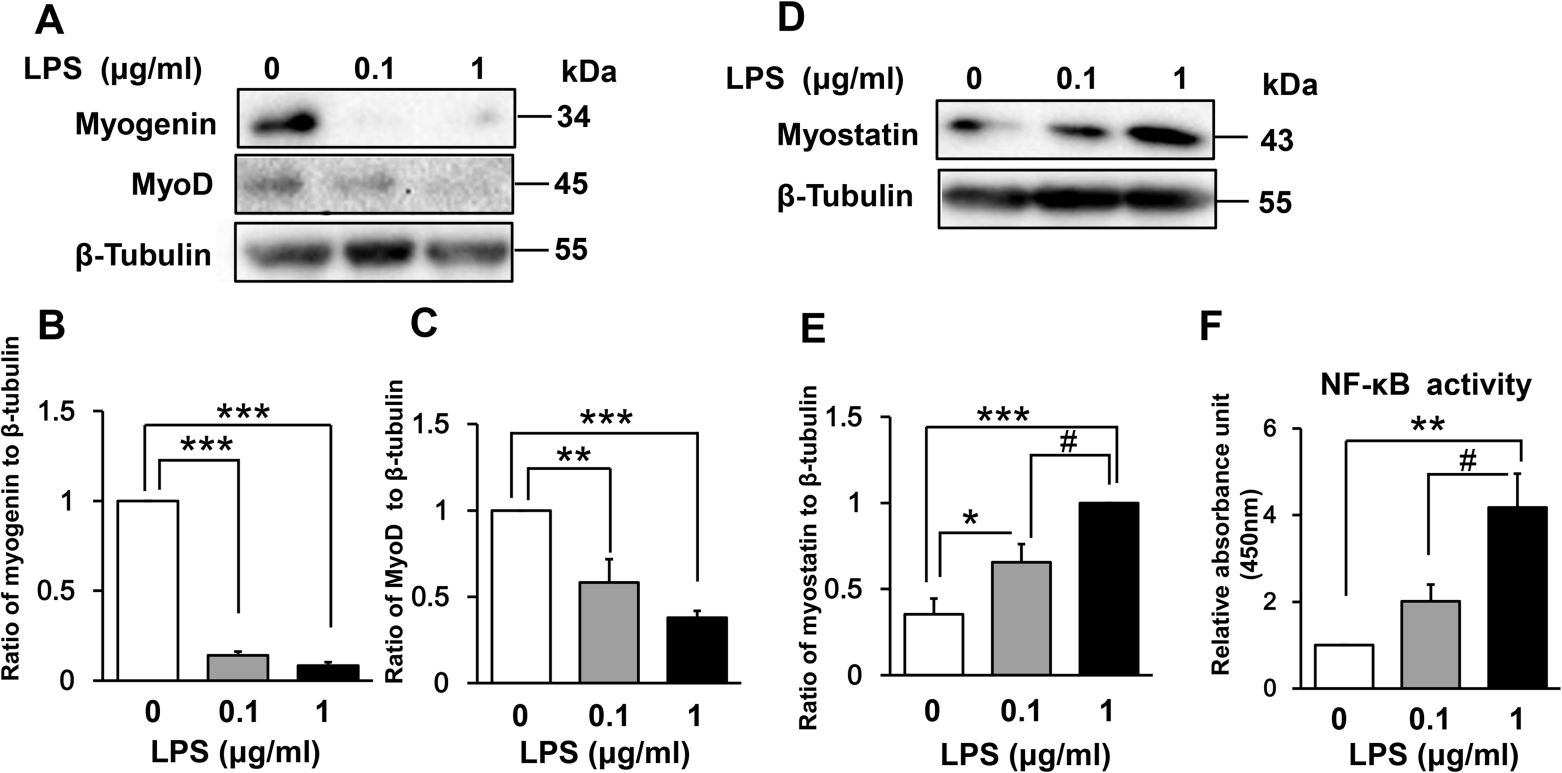
Effect of LPS on muscle regulatory factors and NF-κB activity in differentiating myoblasts. (A) LPS downregulates expression of the positive myogenic regulatory factors myogenin and MyoD in a dose-dependent manner. C2C12 myoblasts were incubated in DM with or without LPS (0.1 or 1 μg/mL) for 48 h. A representative western blot probed with antibodies to myogenin, MyoD, or β-tubulin (internal standard) is shown. (B and C) Quantification of the data presented in (A). Data are the mean ± SEM of 6–7 independent experiments. (D) LPS upregulates expression of the negative myogenic regulatory factor myostatin in a dose-dependent manner. C2C12 myoblasts were treated for 144 h as described in (A). A representative western blot probed with antibodies to myostatin or β-tubulin (internal standard) is shown. (E) Quantification of the data presented in (D). Data are the mean ± SEM of 14 independent experiments. (F) LPS increases NF-κB DNA-binding activity in a dose-dependent manner. C2C12 myoblasts were incubated in DM with or without LPS (0.1 or 1 μg/mL) for 48 h, and NF-κB activity was analyzed using a TransAM ELISA kit. Data are the mean ± SEM of 5 independent experiments performed in duplicate. ***p < 0.001, **p < 0.01, *p < 0.05, #p < 0.05 by one-way ANOVA followed by Tukey’s honest significant difference test.

The myogenic regulatory factors myogenin and MyoD are responsible for the induction and maintenance of early-phase muscle differentiation [32,33]. To understand the mechanism of LPS-induced myogenic inhibition, we examined the expression of these regulatory factors in LPS-treated C2C12 myoblasts. As shown in Fig 3A–3C, LPS treatment for 48 h dose-dependently downregulated the expression of both myogenin (LPS 0.1 μg/mL vs control, 86% decrease [p < 0.001]; LPS 1 μg/mL vs control, 92% decrease [p < 0.001]) and MyoD (LPS 0.1 μg/mL vs control, 42% decrease [p < 0.01]; LPS 1 μg/mL vs control, 62% decrease [p < 0.001]).

We next examined whether LPS-induced inhibition of myogenesis is associated with increased expression of myostatin, which is a critical autocrine and paracrine inhibitor of skeletal muscle growth and differentiation [34–36]. Indeed, myostatin expression was increased dose dependently by incubation with LPS for 144 h (LPS 0.1 μg/mL vs control, 185% increase [p < 0.05]; LPS 1 μg/mL vs control, 283% increase [p < 0.001]; Fig 3D and 3E).

Previous studies have suggested that NF-κB mediates both the downregulation of MyoD and myogenin and the upregulation of myostatin [27–31,37,38]. Thus, we next evaluated NF-κB (p65) DNA-binding activity in LPS-treated myoblasts. C2C12 nuclear extracts were prepared after 48 h of LPS treatment and analyzed by ELISA. This time point was chosen for convenience because the LPS-containing medium was refreshed every other day. As shown in Fig 3F, LPS treatment dose-dependently increased NF-κB DNA-binding activity (LPS 0.1 μg/mL vs control, 201% increase [p = 0.37]; LPS 1 μg/mL vs control, 417% increase [p < 0.01]). Collectively, these data suggest that LPS-induced MyoD and myogenin downregulation and myostatin upregulation is associated with increased NF-κB activity.

### The inhibitory effect of LPS on myogenesis is reversible

**Fig 4:**
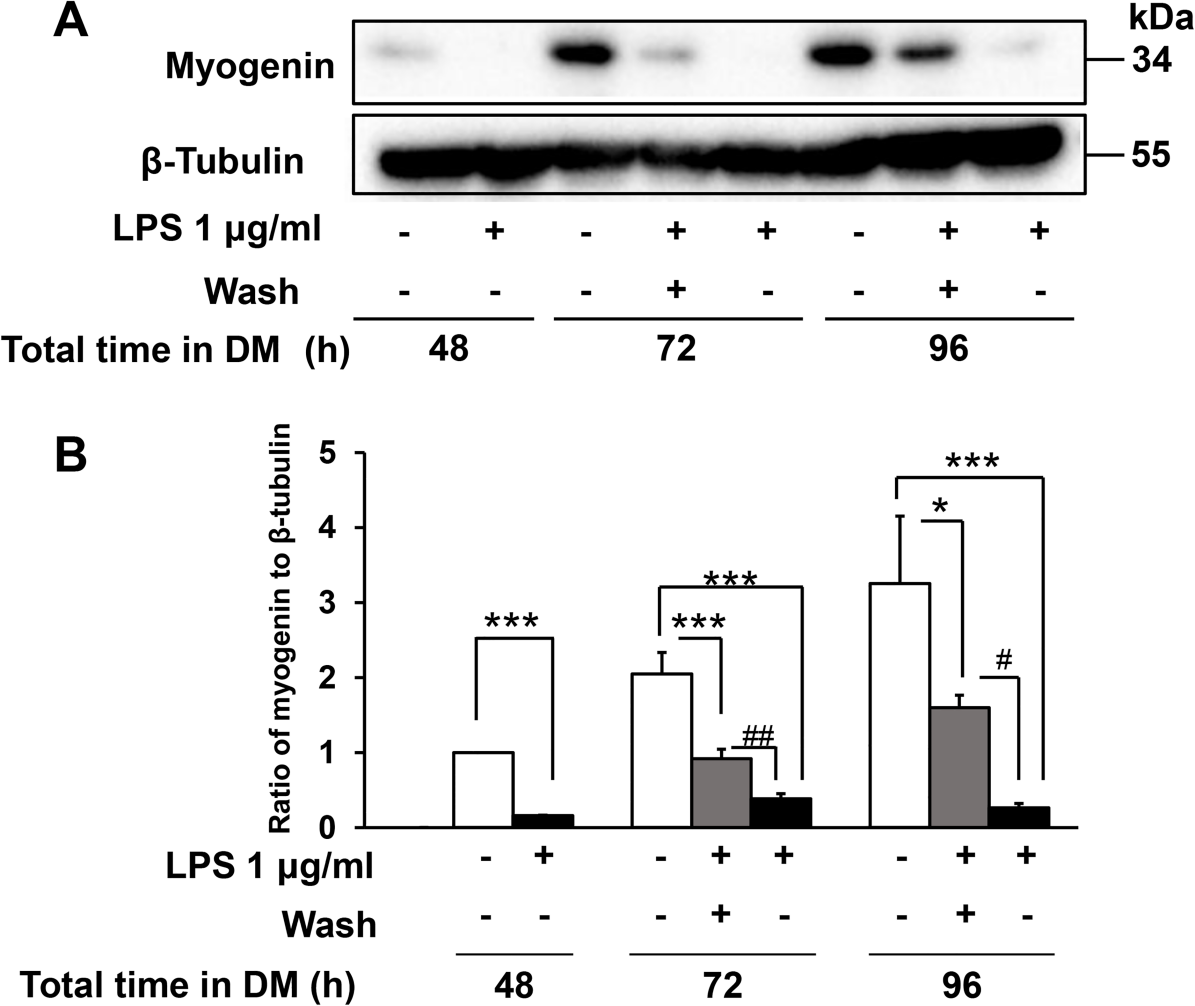
LPS-induced inhibition of myogenin expression is reversible. C2C12 cells were cultured in DM with or without LPS (1 μg/mL) for 48 h, washed free of LPS, and then cultured for an additional 24 h (72 h total) or 48 h (96 h total) in fresh DM. (A) Representative western blot probed with antibodies to myogenin or β-tubulin (internal standard). (B) Quantification of the data presented in (A). Data are the mean ± SEM of 5 independent experiments. ***p < 0.001,*p < 0.05, #p < 0.05, ##p < 0.01 by one-way ANOVA followed by Tukey’s honest significant difference test.

Next, we investigated whether the inhibitory effect of LPS on myogenesis was reversible. To address this question, we adapted the methodology of Langen et al. [28], who showed that the toxic effect of TNF-α on C2C12 myogenesis was reversible. The cells were incubated with LPS for 48 h, washed, and cultured in fresh LPS-free DM. Myogenin expression was examined at 24 and 48 h after LPS washout. Notably, while myogenin expression was initially reduced by LPS, it was restored after washing and removal of LPS (Fig 4A and 4B). These results demonstrate that the inhibitory effect of LPS on myogenic differentiation is reversible, suggesting that it is not due to a nonspecific toxic effect such as induction of cell death.

### Effect of TAK-242 on LPS-induced inhibition of myogenesis

**Fig 5:**
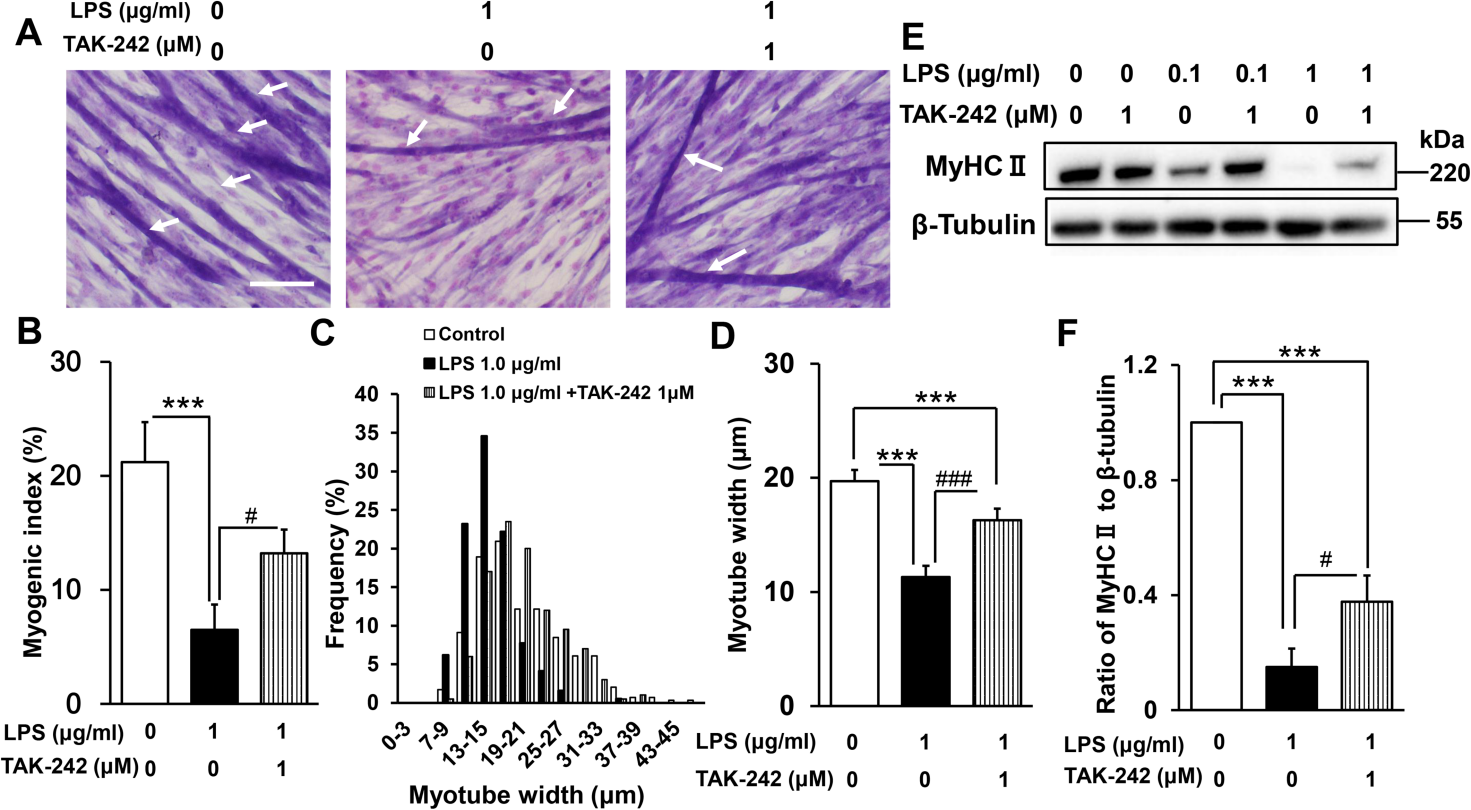
Effect of TAK-242 on LPS-induced inhibition of myogenesis. (A) May–Grünwald–Giemsa staining of C2C12 cells cultured for 144 h in DM alone, DM plus LPS (1 μg/mL), or DM plus LPS (1 μg/mL) and TAK-242 (1 μM). Representative images are shown. Scale bar = 100 μm. Arrows indicate differentiated myotubes. (B) TAK-242 partially reversed the LPS-induced decrease in myogenic index. Cells were treated as described in (A). Data are the mean ± SEM of 10 independent experiments per treatment group, each examining 5 randomly selected fields (total 50 fields). (C and D) TAK-242 partially ameliorated the LPS-induced myotube atrophy. Cells were treated as described in (A), and the distribution of myotube widths (C) and mean myotube widths (D) were calculated for each treatment group. Data are the mean ± SEM of 10–12 independent experiments, each examining 5 randomly selected fields (total 194–296 myotubes from 50–60 fields per treatment group). (E) Representative western blot probed with antibodies to MyHC II or β-tubulin (internal standard). Cells were treated as described in (A). (F) Quantification of the data presented in (E). Data are the mean ± SEM of 12–13 independent experiments. ***p < 0.001, ###p < 0.001, #p < 0.05 by one-way ANOVA followed by Tukey’s honest significant difference test.

We next assessed whether pharmacological inhibition of TLR4 signaling can ameliorate the harmful effect of LPS on C2C12 myogenesis. Myoblasts were incubated for 144 h in the presence or absence of LPS (1 μg/mL) and/or TAK-242 (1 μM), a small molecule specific inhibitor of TLR4 signaling [58,59]. This concentration of TAK-242 was previously found to have a beneficial effect on LPS-induced insulin resistance in L6 myoblast cells [60] and primary human skeletal muscle cells [61]. We found that TAK-242 increased the abundance of multinuclear myotubes in LPS-treated C2C12 cells (myogenic index: LPS 1 μg/mL, 7 ± 2% vs LPS 1 μg/mL + TAK-242 1 μM, 13 ± 2%, p < 0.05; Fig 5A and 5B) as well as myotube width (LPS 1 μg/mL, 11 ± 0.3 μm vs LPS 1 μg/mL + TAK-242 1 μM, 16 ± 0.4 μm, p < 0.001; Fig 5C and 5D). Moreover, TAK-242 partially restored MyHC II expression (LPS 1 μg/mL + TAK-242 1 μM vs LPS 1 μg/mL, 251% increase, p < 0.05; Fig 5E and 5F), confirming its beneficial effect. Higher TAK242 concentrations (10 μM and 100 μM) did not further improve myogenesis and, in fact, were toxic to the cells (data not shown). Collectively, these results indicate that TLR4 signaling is responsible, at least in part, for the inhibition of myogenesis by LPS, since TAK-242 treatment attenuated the effect.

### TLR4–NF-κB signaling mediates LPS-induced dysregulation of muscle regulatory factors

**Fig 6:**
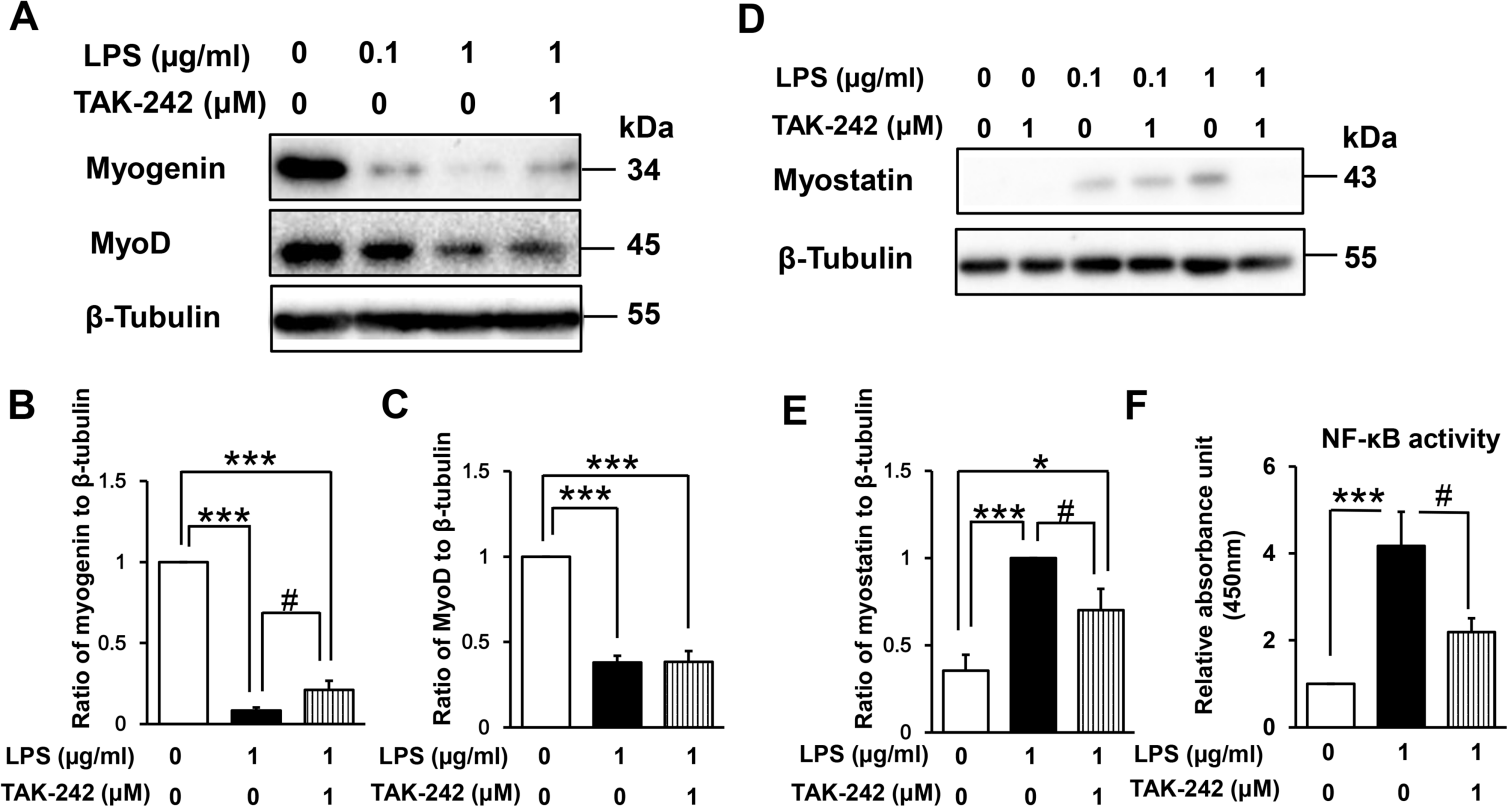
Effect of TAK-242 on LPS-induced perturbation of muscle regulatory factors and NF-κB activity. (A) TAK-242 partially reverses the LPS-induced downregulation of myogenin, but not of MyoD. C2C12 myoblasts were cultured for 48 h in DM alone, with LPS (1 μg/mL or 0.1 μg/mL), or with a combination of LPS (1 μg/mL) and TAK-242 (1 μM). A representative western blot probed with antibodies to myogenin, MyoD, or β-tubulin (internal standard) is shown. (B and C) Quantification of the data presented in (A). Data are the mean ± SEM of 5–7 independent experiments. (D) TAK-242 partially reverses the LPS-induced upregulation of myostatin expression. C2C12 myoblasts were cultured for 144 h as described in (A). A representative western blot probed with antibodies to myostatin or β-tubulin (internal standard) is shown. (E) Quantification of the data presented in (D). Data are the mean ± SEM of 14–16 independent experiments. (F) TAK-242 inhibits NF-κB activity in LPS-treated differentiating myoblasts. Cells were treated as described in (A), and NF-κB activity was analyzed using a TransAM ELISA kit. Data are the mean ± SEM of 5 independent experiments performed in duplicate. ***p < 0.001, *p < 0.05, #p < 0.05 by one-way ANOVA followed by Tukey’s honest significant difference test.

Next, we evaluated whether TLR4 signaling mediates the LPS-induced perturbation of muscle regulatory factors. As shown in Fig 6A–6C, co-administration of TAK-242 (1 μM) partially reversed the LPS-induced decrease in expression of myogenin (LPS 1 μg/mL + TAK-242 1 μM vs LPS 1 μg/mL, 251% increase; p < 0.05) but not of MyoD (101% increase, p = 1.0). Similarly, LPS-induced upregulation of myostatin was partially blocked by TAK-242 (30% decrease vs LPS 1 μg/mL, p < 0.05; Fig 6D and 6E). However, at 0.1 μg/mL LPS, TAK-242 failed to rescue the upregulation of myostatin expression (5% decrease, p = 0.7). TAK-242 also decreased NF-κB DNA-binding activity in LPS-treated myoblasts (48% decrease vs LPS 1 μg/mL, p < 0.05; Fig 6F). These data therefore suggest that TLR4 acts as an upstream regulator of NF-κB-mediated dysregulation of myogenic regulatory factors.

### LPS signals through both TLR2 and TLR4 to activate NF-κB

**Fig 7:**
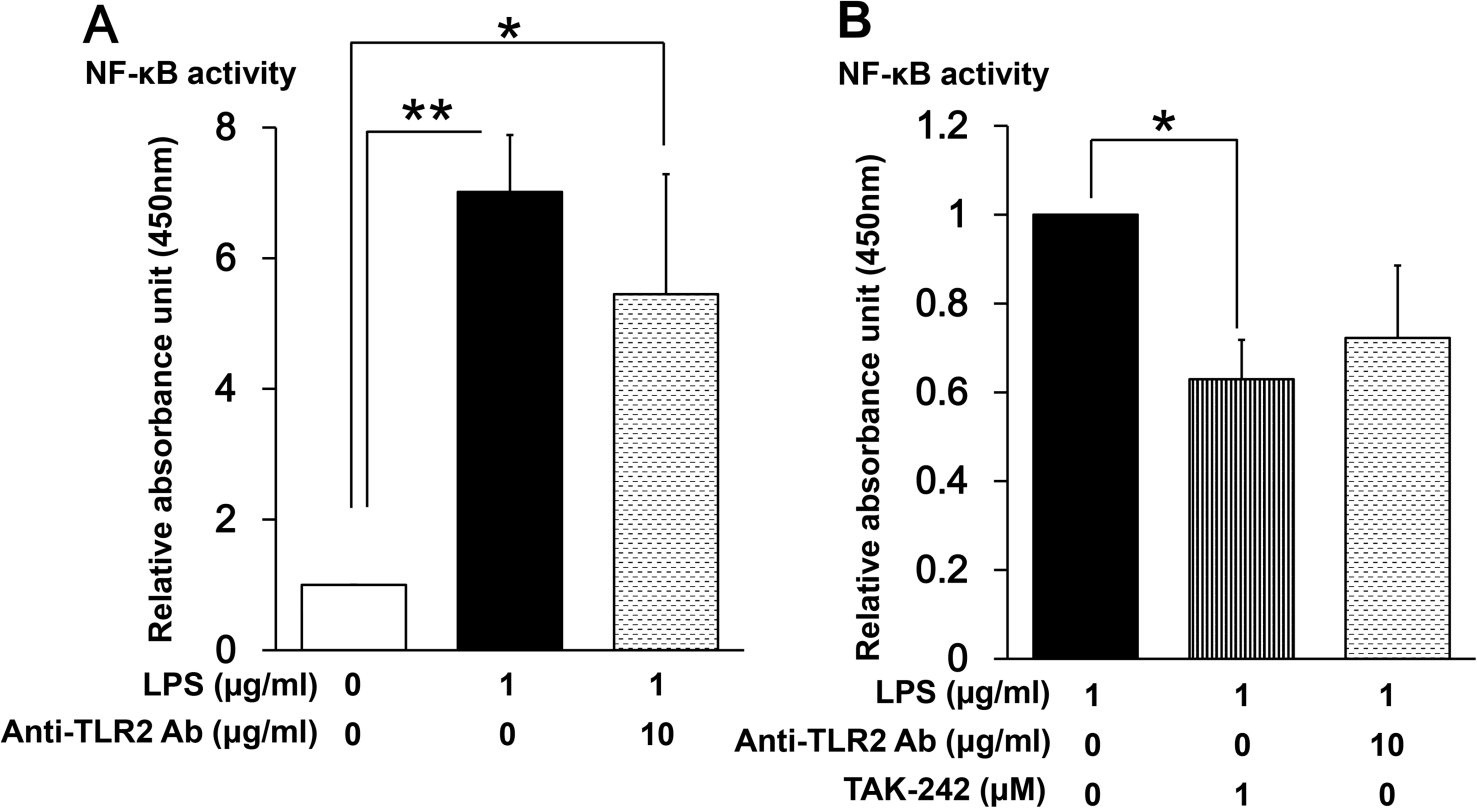
Effect of a TLR2-neutralizing antibody on NF-κB activity in LPS-treated myoblasts. (A) C2C12 myoblasts were cultured for 48 h in DM alone, LPS (1 μg/mL), or LPS (1 μg/mL) plus TLR2-neutralizing antibody (10 μg/mL). Data are the mean ± SEM of 7 independent experiments performed in duplicate. (B) C2C12 myoblasts were cultured for 48 h in DM with LPS (1 μg/mL), LPS (1 μg/mL) plus TAK-242 (1 μM), or LPS (1 μg/mL) plus TLR2-neutralizing antibody (10 μg/mL). NF-κB activity was measured by ELISA. Data are the mean ± SEM of 7–10 independent experiments performed in duplicate. **p < 0.01, *p < 0.05 by one-way ANOVA followed by Tukey’s honest significant difference test.

As demonstrated in Fig 5 and Fig 6, selective inhibition of TLR4 signaling did not fully suppress LPS-induced NF-κB activation and dysregulation of muscle regulatory factors. Since LPS is also a ligand for TLR2 [62–64], and TLR2 mRNA is expressed in C2C12 myoblasts [41,43], we considered that LPS might be signaling through both TLR2 and TLR4. To examine this, myoblasts were incubated with 1 μg/mL LPS in the presence or absence of a TLR2-neutralizing antibody (T2.5, 10 μg/mL), and NF-κB activity was measured after 48 h. This concentration of anti-TLR2 antibody was previously found to prevent serum amyloid A-induced TLR2-dependent signaling in C2C12 myotubes [56]. As shown in Fig 7A, addition of anti-TLR2 decreased LPS-induced NF-κB activation by about 30%, although the difference did not reach the level of statistical significance (p = 0.62). The selective TLR4 inhibitor had a greater effect on NF-κB activity (Fig 7B), suggesting that LPS signals predominantly through TLR4 but at least partially through TLR2 in these cells, This could be one possible explanation for the failure of TAK-242 to completely suppress the LPS-induced effects on NF-κB and myogenic regulatory factors.

### Myoblast-derived autocrine/paracrine TNF-α is involved in LPS-induced inhibition of myogenesis

**Fig 8:**
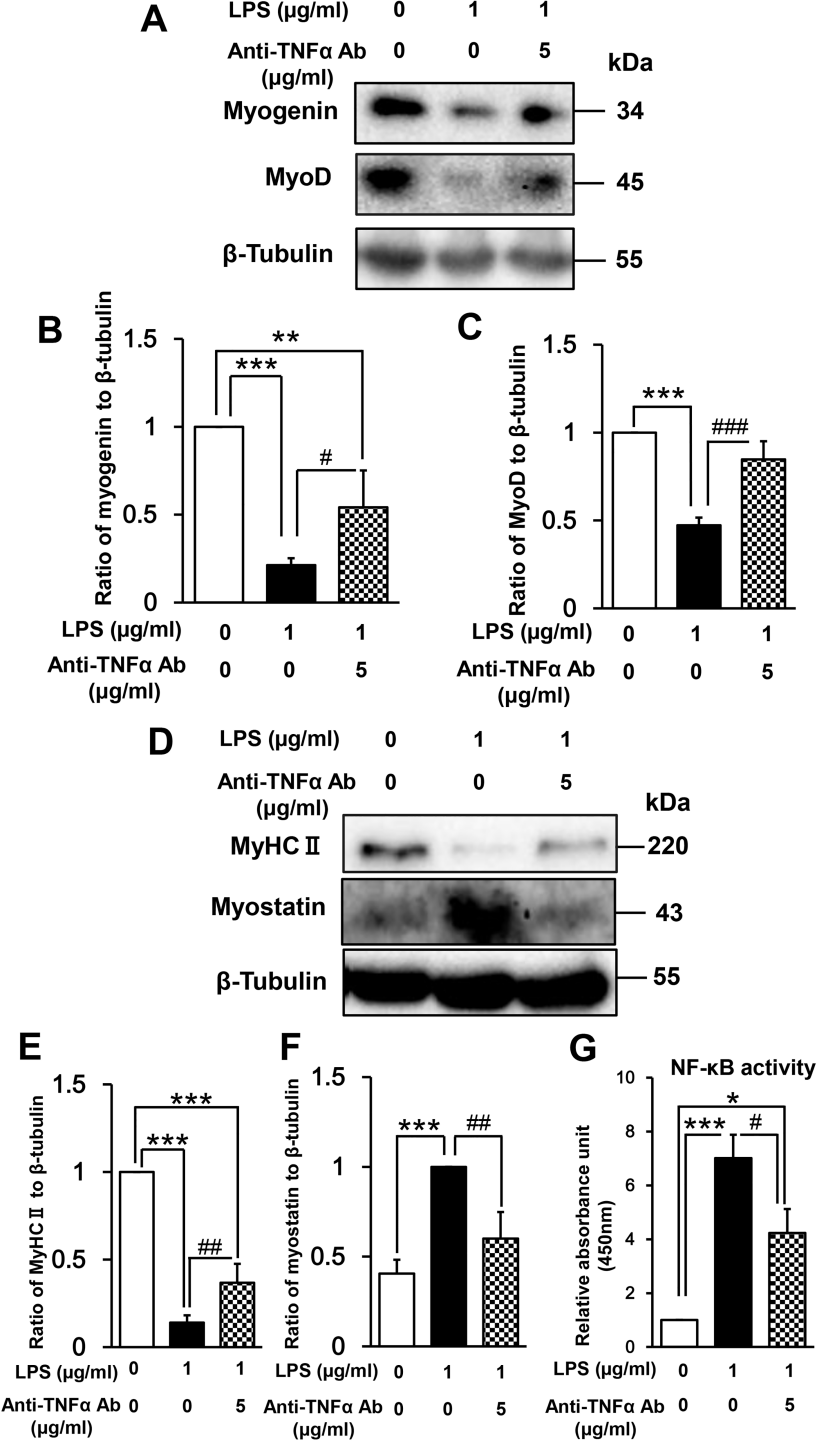
Effect of a TNF-α-neutralizing antibody on LPS-induced perturbation of muscle differentiation. (A) C2C12 myoblasts were cultured for 48 h in DM alone, LPS (1 μg/mL), or LPS (1 μg/mL) plus TNF-α-neutralizing antibody (5 μg/mL). A representative western blot probed with antibodies to myogenin, MyoD, or β-tubulin (internal standard) is shown. (B and C) Quantification of the data presented in (A). Data are the mean ± SEM of 7–15 independent experiments. (D) C2C12 myoblasts were cultured for 144 h as described in (A). A representative western blot probed with antibodies to MyHC II, myostatin, or β-tubulin (internal standard) is shown. (E and F) Quantification of the data presented in (D). Data are the mean ± SEM of 5–21 independent experiments. (G) Cells were treated as described in (A), and NF-κB activity was analyzed by ELISA. Data are the mean ± SEM of 7 independent experiments performed in duplicate. ***p < 0.001, **p < 0.01, *p < 0.05, ###p < 0.001, ##p < 0.01, #p < 0.05 by one-way ANOVA followed by Tukey’s honest significant difference test.

There is considerable evidence that LPS regulates the expression of proinflammatory cytokines, such as TNF-α and interleukin 6, in mouse myoblasts and skeletal muscle [40,41,43], and TNF-α has previously been shown to inhibit myogenic differentiation [27–31]. Therefore, we hypothesized that myoblast-derived TNF-α may be involved in LPS-induced inhibition of myogenesis. To test this hypothesis, we examined C2C12 cells after treatment with LPS (1 μg/mL) in the absence or presence of a TNF-α-neutralizing antibody (5 μg/mL). This concentration of antibody was previously shown to inhibit TNF-α secretion and subsequent NF-κB activation induced by serum restriction of C2C12 myoblasts [65]. We found that the TNF-α-neutralizing antibody partially but significantly reversed the LPS-induced downregulation of myogenin (LPS + anti-TNF-α vs LPS, 255% increase, p < 0.05; Fig 8A and 8B), MyoD (179% increase, p < 0.001; Fig 8A and 8C), and MyHC II (262% increase, p < 0.01; Fig 8D and 8E) expression, and additionally suppressed the LPS-induced upregulation of myostatin expression (40% decrease, p < 0.01; Fig 8D and 8F) and NF-κB activation (40% decrease, p < 0.05; Fig 8G). These results suggest that NF-κB activation and subsequent effects on myogenic regulatory factors induced by LPS are partially mediated by myoblast-derived TNF-α. Thus, it is possible that such an autocrine/paracrine TNF-α loop is involved in the impairment of muscle regeneration.

**Fig 9:**
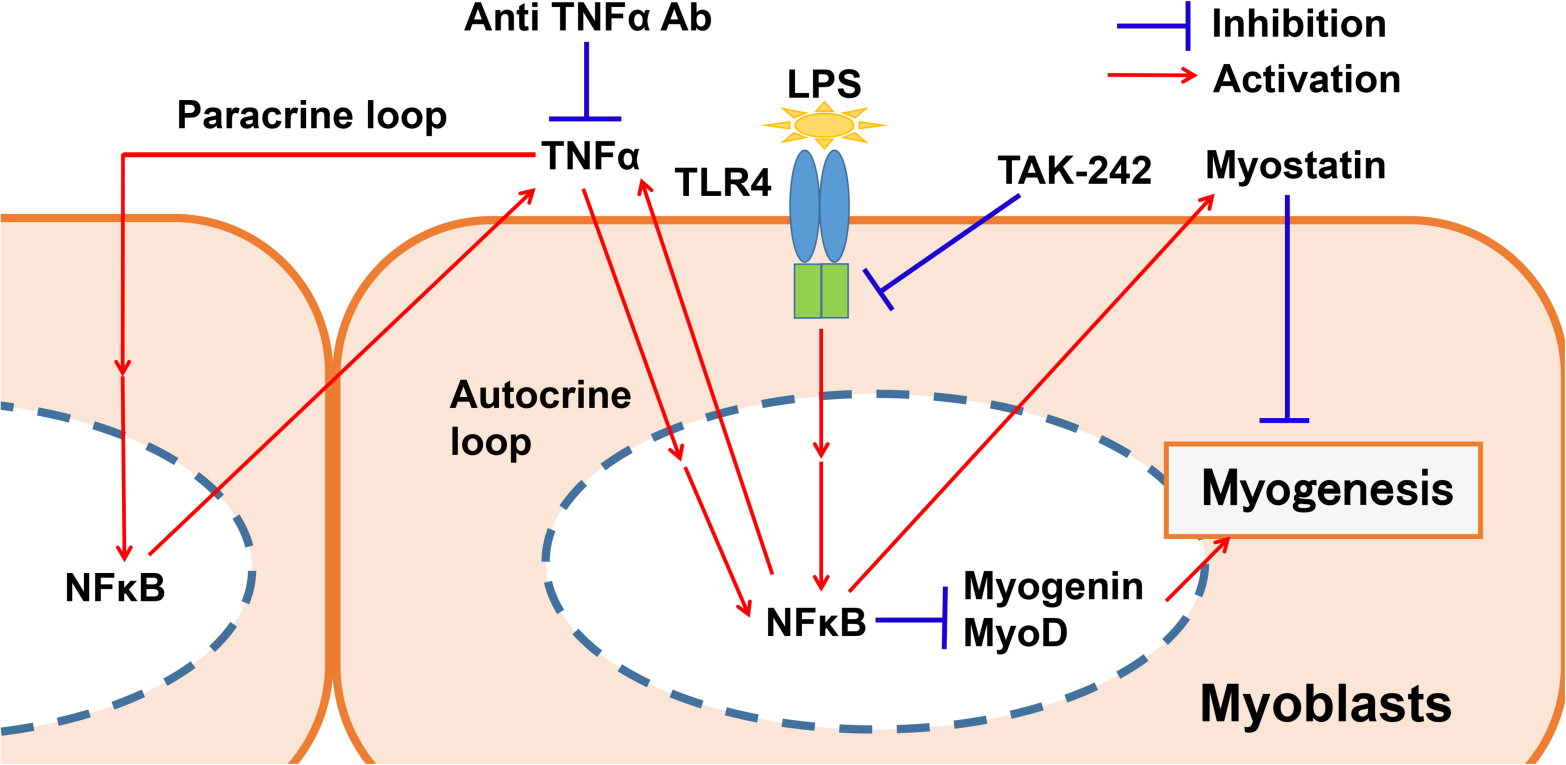
Proposed mechanism of LPS inhibition of myogenesis in differentiating myoblasts.

Based on these collective data, we propose a mechanistic model for the inhibition myogenesis by LPS in differentiating myoblasts (Fig 9).

## Discussion

In the present study, we found that LPS inhibits C2C12 myogenesis through the TLR4–NF-κB and autocrine/paracrine TNF-α-mediated pathways. We found that LPS downregulated MyoD and myogenin expression and upregulated myostatin expression in a dose-dependent manner. Both pharmacological inhibition of TLR4 signaling and antibody-mediated neutralization of TNF-α reduced NF-κB activity and attenuated the LPS-induced dysregulation of muscle regulatory factors.

For our in vitro experiments, we employed two concentrations of LPS, 0.1 and 1 μg/mL, and observed a dose-dependent inhibitory effect on murine myoblast differentiation. A previous study suggested that humans were more than 10,000-fold more sensitive than mice to LPS [66], raising the possibility that human muscle regeneration could be much more vulnerable to the effects of LPS. Circulating LPS levels are commonly elevated in conditions such as sepsis [4] and endogenous diseases [5–15], and muscle atrophy can also be observed in these conditions [16]. Therefore, LPS-induced derangement of myogenesis might be a cause of muscle wasting in patients with sepsis or metabolic endotoxemia.

In this study, we demonstrated that the TLR4 signaling pathway mediated LPS-induced activation of NF-κB, downregulation of MyoD and myogenin expression, and upregulation of myostatin expression. Our findings are in agreement with several earlier observations that exogenous TNF-α-induced activation of NF-κB inhibited myogenesis in C2C12 cells by suppressing MyoD and myogenin expression [27–31]. NF-κB-mediated upregulation of myostatin has also been observed in other model systems, such as H_2_O_2_-treated cultured myoblasts [38] and mouse models of liver cirrhosis and hyperammonemia [37]. In contrast, several studies have suggested that myostatin expression in skeletal muscle is not increased in sepsis models. For example, Smith et al. observed that myostatin mRNA levels were reduced and myostatin protein levels were unchanged in rat skeletal muscle 16 h after cecal ligation and puncture [67]. Lang et al. reported that myostatin mRNA was not increased 24 h after LPS administration to rats [68]. One possible explanation for this discrepancy is the shorter endotoxin exposure times, since we observed increased myostatin expression in C2C12 cells after 144 h of LPS treatment. Martin et al. noted a time-dependent increase in myostatin mRNA expression after administration of LPS to mice; they found that the levels remained unchanged at 24 h after LPS injection but increased significantly after 76 h [69]. In the clinical setting, sepsis survivors often display systemic inflammation for protracted periods and also develop muscle wasting. Circulating myostatin is commonly elevated in patients with conditions such as chronic liver and kidney disease [25,37,70], diabetes mellitus [71], and human immunodeficiency virus infection [72], and in the elderly [73,74]. All of these populations are likely to be chronically exposed to LPS due to bacterial translocation [15]. Taken together, these findings suggest that persistent exposure to LPS or inflammation may be required to induce myostatin.

We found that myogenin expression was rapidly restored after switching from LPS (1 μg/mL)-containing medium to fresh medium, suggesting that the inhibition of myogenesis was reversible and not simply a toxic effect of LPS, such as induction of apoptosis. Shang et al. examined C2C12 cell viability after exposure to various concentrations of LPS [75]. They found that LPS at 1–10 μg/mL had no effect on C1C12 apoptosis, whereas higher concentrations (100–150 μg/mL) induced apoptosis through caspase-3 activation. Our findings are thus consistent with their data. Reversible inhibition of myogenesis has also been observed upon treatment of C2C12 myoblasts with TNF-α [28]. Taken together, these data suggest that persistent NF-κB activation may be required to block myogenesis and promote muscle wasting.

While the role of TLR4 in innate immunity is well characterized, its role in skeletal muscle development has been unclear. To address this knowledge gap, we examined the effect of a selective TLR4 signaling pathway inhibitor on the LPS-induced events. We observed that TAK-242 partially rescued the LPS-induced inhibition of myogenesis, activation of NF-κB, downregulation of myogenin, and upregulation of myostatin, suggesting that TLR4 is an upstream regulator of skeletal muscle myogenesis. Previous studies have suggested that TLR4 plays an important role in muscle protein breakdown. Doyle et al. [19] found that TLR4-mediated LPS signaling induced muscle catabolism via coordinate activation of the ubiquitin–proteasome and autophagy pathways. According to Dehoux et al. [20] and Martin et al. [69], ubiquitin ligase mRNA expression was induced in both rat and mouse skeletal muscle after LPS injection. In agreement with these findings, we observed that LPS dose-dependently decreased the myotube width, and that inhibition of TLR4 signaling prevented the atrophy. Collectively, these findings indicate that LPS may induce muscle wasting via synergistic effects on myogenesis and muscle proteolysis through TLR4. Skeletal muscle TLR4 is upregulated in diabetic and obese subjects [76] as well as in the elderly [5,77], suggesting that the TLR4–NF-κB pathway may be elevated in these populations. Thus, inhibition of the TLR4 signaling axis might be a useful method for preventing or reversing LPS-induced muscle wasting in patients with sepsis or metabolic endotoxemia. Future studies should address the effects of TLR4 antagonists on LPS-induced muscle breakdown.

We found that antibody-mediated neutralization of TNF-α reduced the LPS-induced increase in NF-κB DNA-binding activity, downregulation of myogenin and MyoD, and upregulation of myostatin in C2C12 cells, suggesting that LPS-induced autocrine/paracrine TNF-α might be involved in the impairment of muscle regeneration. Autocrine/paracrine regulation of TNF-α has also been observed in C2C12 myoblasts following serum restriction [65] and in various other cell lines and tissues, including cancer cells [46,78], immune cells [79,80], and microglia [81]. In skeletal muscle myoblasts, TNF-α is a strong activator of NF-κB [65] and of its own synthesis [82]. Therefore, as is the case in cancer cells [46,78], a positive TNF-α autocrine/paracrine loop in response to LPS may lead to persistent NF-κB activation in myoblasts, further inhibiting myogenesis and thus inducing muscle wasting.

Interestingly, we found that TNF-α neutralization, but not TLR4 inhibition by TAK-242, reversed the LPS-induced inhibition of MyoD expression. We speculate that MyoD expression might be more sensitive to regulation by TNF-α than by TLR4 signaling. In support of this possibility, the pattern of NF-κB activation by LPS and TNF-α has been shown to differ. In C2C12 myotubes, TNF-α was found to persistently activate NF-κB in a biphasic manner, while LPS did not [31]. Moreover, in human epithelial cells, LPS from *Haemophilus influenzae* (exogenous activation) and TNF-α (endogenous activation) synergistically induced NF-κB activation via two distinct signaling pathways [83]. In addition to the involvement of TLR2 mediated signaling, this could be another explanation for the failure of TAK-242 to fully ameliorate the harmful effect of LPS on differentiating myoblasts.

In vivo, LPS induces circulating immune cells to produce copious amounts of inflammatory cytokines, which have been implicated as potential mediators of muscle wasting via inhibition of myogenesis [27–31] and acceleration of muscle proteolysis [18,84,85]. In fact, inflammatory cytokine concentrations are elevated in the circulation of patients with sepsis [86] and metabolic endotoxemia [87–89]. Our study extends these observations by demonstrating that LPS itself can directly inhibit myogenesis through TLR4–NF-κB signaling and myoblast-derived TNF-α. Systemic and local inflammatory reactions may synergize to induce muscular wasting.

In this study, cells were co-incubated with TAK-242 dissolved in DMSO (final concentration 0.1% [vol/vol]) and LPS dissolved in PBS. We acknowledge that a DMSO vehicle control was not included in two experiments shown in Figs 5 and 6. High concentrations of DMSO (1–2% [vol/vol]) have been shown to augment the LPS effect on immune cells (i.e., increase inflammatory cytokine secretion) [90]; therefore, in our study, the relative effect of TAK-242 on the LPS response may have been weakened. Nevertheless, previous studies have shown that, even at concentrations as high as 1–2% (vol/vol), DMSO has no effect on NF-κB activity in various cell lines, reducing this concern [90–92].

We observed that TAK-242 significantly decreased myostatin expression when cells were stimulated with LPS at 1 μg/mL (30% decrease, p < 0.05) but not at 0.1 μg/mL (5% decrease, p = 0.7). At present, we do not have a plausible explanation for this discrepancy.

To date, no drugs have been approved for the treatment of skeletal muscle wasting. Our finding that blockade of the TLR4–NF-κB pathway or TNF-α can reverse impaired myogenesis suggests a new set of drug targets for clinical intervention in sepsis- or metabolic endotoxemia-induced muscle debilitation. Clinical trials with a TLR4 antagonist [93–95] and TNF-α inhibitor [96] have shown no improvement of the mortality rate of severe sepsis patients; however, those trials did not examine long-term muscle function [93–96]. The results presented here provide a rationale to test the effects of TLR4 and TNF-α antagonists on LPS-induced muscle wasting in sepsis or metabolic endotoxemia patients. Our data should also stimulate further studies to clarify the role of TLR4–NF-κB and TNF-α signaling in muscle wasting.

## Conclusions

LPS inhibits C2C12 myogenesis by dose-dependently downregulating myogenin and MyoD expression and upregulating myostatin expression. TAK-242, a selective inhibitor of TLR4-mediated signaling, and a TNF-α-neutralizing antibody both reduced NF-κB activity and attenuated the downregulation of myogenic regulatory factors and upregulation of myostatin in LPS-treated C2C12 myoblasts. Consequently, myogenesis was partially restored. These data suggest that LPS inhibits myogenic differentiation of skeletal muscle myoblasts through TLR4–NF-κB signaling and myoblast-derived autocrine/paracrine TNF-α, raising the possibility that these pathways also contribute to the development of muscle wasting in patients with sepsis or metabolic endotoxemia.

## Supporting information

S1 Data Excel spreadsheet providing the raw data for each figure. (XLSX)
